# Evaluation of Methods for Identifying Human Monocytes by Flow Cytometry: TLR2 May be an Optimal Marker

**DOI:** 10.1111/imm.70195

**Published:** 2026-09-07

**Authors:** Mie Wolff Kristensen, Katrine Skjøt Harbo, Thomas Vorup‐Jensen, Marianne Hokland, Morten Nørgaard Andersen

**Affiliations:** ^1^ Department of Biomedicine Aarhus University Aarhus Denmark; ^2^ Department of Hematology Aarhus University Hospital Aarhus Denmark; ^3^ Department of Clinical Medicine Aarhus University Aarhus Denmark; ^4^ Department of Molecular Medicine (MOMA) Aarhus University Hospital Aarhus Denmark

**Keywords:** flow cytometry, monocytes, peripheral blood mononuclear cells, TLR2

## Abstract

Monocytes play crucial roles in the pathobiology of a wide range of medical conditions, including infection, autoimmunity, cardiovascular disease, and malignancies. Studies using flow cytometry revealed the complexity of monocyte biology, including identification of three human monocyte subsets: classical (CD14^pos^ CD16^neg^), intermediate (CD14^pos^ CD16^pos^) and non‐classical (CD14^neg/dim^ CD16^pos^) monocytes. Still, gating strategies used for identification of monocytes by flow cytometry vary substantially between studies, which may significantly impact results and hamper reproducibility. However, to our knowledge, no previous study has evaluated several different gating strategies for identification of the total circulating monocyte population in humans. Here, healthy donor peripheral blood mononuclear cells (PBMCs, *n* = 10) were analysed by flow cytometry to compare seven commonly used gating strategies for defining human monocytes: (1) negative selection (CD3^neg^ CD19^neg^ CD56^neg^ CD66^neg^), (2) FSC‐SSC, (3) CD14^pos^, (4) CD64^pos^, (5) HLA‐DR^pos^, (6) CD14—HLA‐DR and (7) TLR2^pos^. We show that the different gating strategies significantly impacted the enumeration of non‐classical monocytes, but less so for classical and intermediate monocyte populations. Compared to negative selection gating (considered the reference standard), the FSC‐SSC‐based gating showed the highest degree of contamination by other cell types, whereas CD14^pos^, CD64^pos^, CD14‐HLA‐DR and HLA‐DR^pos^ gating showed highest degree of exclusion of monocytes, particularly non‐classical monocytes. In comparison, TLR2^pos^ gating showed both the least contamination and least erroneous exclusion of monocytes when compared to negative selection gating. These results based on ‘manual gating’ were supported by more objective t‐SNE and clustering analyses. In conclusion, TLR2^pos^ gating and negative selection‐based gating displayed both minimal contamination and exclusion of true monocytes, with TLR2^pos^ gating yielding the overall purest monocyte population. Thus, adapting to more uniform gating strategies for monocyte analysis, such as a TLR2 based gating strategy, may increase data quality and reproducibility between laboratories.

## Graphical abstract

Monocytes play crucial roles in the pathobiology of a wide range of medical conditions, including infection, autoimmunity, cardiovascular disease and malignancies. Studies using flow cytometry revealed the complexity of monocyte biology, including the identification of three human monocyte subsets: classical (CD14^pos^ CD16^neg^), intermediate (CD14^pos^ CD16^pos^) and non‐classical (CD14^neg/dim^ CD16^pos^) monocytes. Nevertheless, the gating strategies used for the identification of monocytes by flow cytometry vary substantially between studies, which may significantly impact results and hamper reproducibility. We show that the different gating strategies significantly impacted the enumeration of non‐classical monocytes, but less so for classical and intermediate monocyte populations. In conclusion, TLR2^pos^ gating and negative selection‐based gating displayed both minimal contamination and exclusion of true monocytes, with TLR2^pos^ gating yielding the overall purest monocyte population. Adapting to more uniform gating strategies for monocyte analysis, such as a TLR2‐based gating strategy, may increase data quality and reproducibility between laboratories.

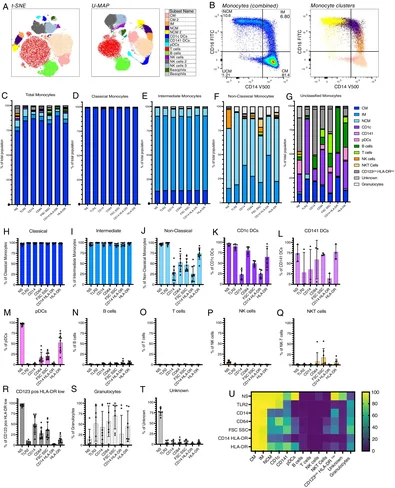

**Abbreviations**
CyTOF: cytometry by time of flight
DCs: dendritic cells
DMSO: dimethylsulfide
FCS: foetal calf serum
FSC: forward scatter
MGUS: monoclonal gammopathy of unknown significance
PBMCs: peripheral blood mononuclear cells
PBS: phosphate buffered saline
SSC: side scatter
TLR2: toll‐like‐receptor 2
t‐SNE: t‐distributed stochastic neighbour embedding
UMAP: uniform manifold approximation and projection

## 1 Introduction

Monocytes are immune cells of the innate immune system that can transmigrate from the blood into tissues and may differentiate into macrophages [1, 2]. Awareness of the pathophysiological significance of monocytes, in, for example, coronary artery disease, several malignancies, infections, and autoimmune diseases, has increased over the past decades, mainly through flow cytometry analyses of peripheral blood monocyte subsets in patients and healthy donors [2, 3, 4, 5, 6, 7].

Since the late 1980's, flow cytometry has enabled identification of different monocyte subsets based on their expression of CD14 (LPS receptor) and CD16 (Fc‐γ receptor III) [8, 9]. This led to classification of ‘classical’ (CD14^pos^ CD16^neg^), ‘Intermediate’ (CD14^pos^ CD16^neg^) and ‘non‐classical’ (CD14^neg/dim^ CD16^pos^) monocytes [2]. Non‐classical monocytes are described as CD14 dim or negative [10], and will be referred to as dim/neg in this article. Further, when gating monocyte subsets, a double negative (CD14^dim/neg^ CD16^neg^) population is also evident, although often discarded from further analysis. We originally termed this population ‘unclassified monocytes’ but have now shown (in non‐peer‐reviewed data) that within TLR2^pos^ cells, it likely consists mainly of CD1c^pos^ CD163^pos^ dendritic cells (DCs). (Kristensen et al., BIORXIV https://www.biorxiv.org/content/10.64898/2026.07.21.739900v1).

In the literature, gating strategies used to define human monocytes by flow cytometry vary tremendously, making comparisons between studies difficult [2]. However, no study has evaluated which of several widely used gating strategies most accurately defines the ‘total peripheral blood monocyte population’ by flow cytometry.

In general, the strategies used for identification of monocytes by flow cytometry comprise (1) forward scatter (FSC) versus side scatter (SSC) gating, (2) negative selection and (3) positive selection [2, 11]. Monocytes can be defined based on light‐scatter measurements, as FSC^high^ SSC^high^ cells in samples of peripheral blood mononuclear cells (PBMCs) and FSC^high^ SSC^int^ in whole blood samples [2]. Importantly, Ziegler‐Heitbrock and colleagues argued that FSC‐SSC gating is highly subjective and increase the risk of excluding especially intermediate monocytes, while including some activated T cells [2, 12]. It may also be problematic for quantification of especially non‐classical monocytes if the sample includes NK cells or granulocytes, which are also CD14^neg^ CD16^pos^ [12].

Traditionally, negative selection gating has been considered the ‘gold standard’ for monocyte identification by some researchers [2, 10], for example, by removing T cells (CD3^pos^), B cells (CD19^pos^ or CD20^pos^) and NK cells (CD56^pos^) to study monocytes in a PBMC sample. Thus, in the present study, negative selection was used as the ‘reference standard’. However, compared to positive selection, negative selection is more expensive in terms of antibody consumption, results in more complex antibody panels and, due to nonspecific staining, also carries the risk of erroneously excluding monocytes [13, 14]. Finally, whereas negative selection may be feasible in relatively simple samples such as PBMCs, more complex samples (e.g., tumour tissue) may require several additional markers for proper exclusion, potentially making a strategy with positive selection of monocytes more attractive.

Still, no single ideal marker has been widely accepted for defining the total monocyte population of PBMCs [2, 15]. In the literature, various surface markers have been used to define monocytes by positive selection, for example, CD14 [16, 17], TLR2 [15], HLA‐DR [18] and CD64 [19], but separation between negative and dim populations might be difficult, and the expression of these markers, especially HLA‐DR and CD14, can vary depending on disease status [15, 20, 21].

Based on work by Shirk et al. [15], TLR2 was identified as a potentially useful single marker for gating human monocytes by flow cytometry, which could allow for a simple and reproducible gating strategy for defining ‘the total human monocyte population’. However, only few studies have used this method, and thus knowledge is still limited.

Here, we compared seven different gating strategies, including both negative and positive selection, to investigate advantages and disadvantages when aiming to define the ‘total monocyte population’ as well as monocyte subpopulations in healthy human PBMC samples. We conclude that, in our hands, a TLR2‐based positive selection gating strategy most accurately identifies the total monocyte population. Overall, this was also the case for the negative selection‐based strategy, although with a higher degree of contamination by non‐monocytes.

## 2 Materials and Methods

### 2.1 Literature Overview

To provide an overview of the strategies most commonly used to identify human monocytes by flow cytometry, we reviewed articles published over a 9.5‐year period (January 2017 to June 2026). We searched PubMed using the search strategy ‘Flow Cytometry’ AND ‘Monocytes’, identifying 7399 papers, of which 4788 contained work with human cells. These were screened for relevance by title and abstract, and the full text was examined for 1283 papers.

The exclusion criteria were: (1) non‐human‐based work (e.g., animal models), (2) immortalised monocytic cell lines only, (3) non‐peripheral blood analysis only (e.g., tissue‐based work), (4) no clear description of the gating strategy, (5) isolation or sorting of monocytes prior to flow cytometry analysis (e.g., MACS beads), (6) Analysis performed by Mass Cytometry or CyTOF and (7) absence of manual gating (e.g., analysis performed by FlowSOM, X‐shift or similar tool).

Finally, 542 papers were included in the present literature overview. Here, gating strategies were separated into three main categories: (1) FSC‐SSC, (2) negative selection and (3) different positive selection‐based gating strategies (Figure 1).

**FIGURE 1:**
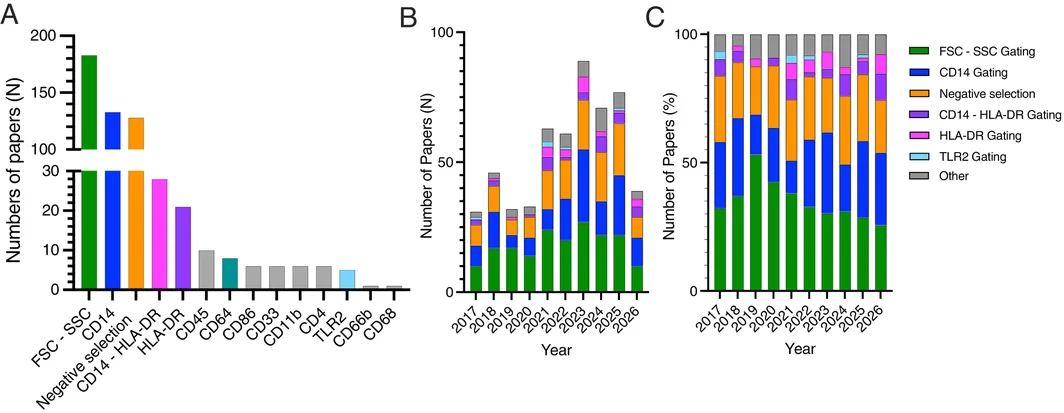
Monocyte gating strategies in the literature. To give an overview of the gating strategies used to identify human monocytes by flow cytometry, we reviewed papers from a 9.5‐year‐period (January 2017–June 2026, *n* = 542). We searched PubMed using the search strategy ‘Flow Cytometry’ AND ‘Monocytes’, excluding non‐human cell‐based work as described in the Materials and Methods section. (A) Number of papers (N), stratified by used gating strategy. (B) Number of papers (N), stratified by both gating strategy and publication year. (C) Percent (%) of papers stratified by both gating strategy and publication year.

### 2.2 Samples

PBMCs were isolated from anonymised healthy blood donor buffy coats (*n* = 10) obtained from the blood bank, Department of Clinical Immunology, Aarhus University Hospital, Aarhus, Denmark (project no. 77) in accordance with Danish law.

### 2.3 Isolation of Mononuclear Cells

The PBMCs were isolated as described previously [13]. Briefly, cells were isolated using density gradient centrifugation on a Histopaque‐1077 gradient (Sigma‐Aldrich, Munich, Germany) according to manufacturer's instructions. After isolation, cells were washed twice in PBS with 2% (v/v) foetal calf serum (FCS, Lonza, Basel, Switzerland) and stored at −150°C in RPMI‐1640 medium with penicillin and streptomycin (100 U/100 μg/mL, Lonza) containing 20% FCS and 10% (v/v) DMSO (Sigma Aldrich). Cells were thawed at 37°C, washed once in PBS with 20% FCS, and resuspended in stain buffer (PBS with 0.5% (w/v) bovine serum albumin (BSA, Calbiochem, San Diego, CA) and 0.09% (w/v) NaN_3_ (Sigma‐Aldrich)), at a final cell concentration of 10 × 10^6^/mL.

Samples were handled as described above and frozen upon arrival by trained laboratory personnel. They were thawed immediately before use in experiments, meaning no samples were exposed to repeated freeze–thaw cycles.

### 2.4 Staining Procedure and Flow Cytometry

The following blocking reagents were added: Fc receptor blocking (100 μg/mL, purified human IgG, Beriglobin) and the phosphorothio‐DNA reagent that we have previously termed ‘Oligo‐Block’ (7.5 μg/mL, 5′‐GGGGGAGCATGCTGGGGGGG‐3′, Sigma‐Aldrich) [13, 14]. The cell suspension was incubated for 15 min at 4°C. Fluorochrome‐conjugated monoclonal antibodies were added (see Table S1), without removing the blocking reagents, and samples were incubated in the dark for 30 min at 4°C, in a final staining volume of 100 μL. After incubation, samples were washed in staining buffer, fixed with 0.9% (v/v) formaldehyde in PBS, washed again and resuspended in staining buffer. The antibody panel was based on Mair et al. [22].

As compensation controls, we used single stained OneComp eBeads (eBiosciences, San Diego, CA) with positive and negative beads for all primary antibodies. Methanol fixed cells were used for 7‐AAD compensation with a positive and negative (unstained) population.

Samples were analysed on a LSRFortessa flow cytometer (BD Biosciences, San Jose, CA). All fluorescence values were recorded as voltage pulse area and all events were within the linear range of the detector. A minimum of 500 000 events were acquired for all PBMC samples and a minimum of 30 000 events for compensation samples. Data were compensated and analysed, and figures were made using FlowJo 10.10 for Mac (FlowJo LLC, Ashland, OR). Figure layout was done in Affinity Designer (Affinity Serif, Nottingham, UK). Gating Strategies are shown in Figure 2.

**FIGURE 2:**
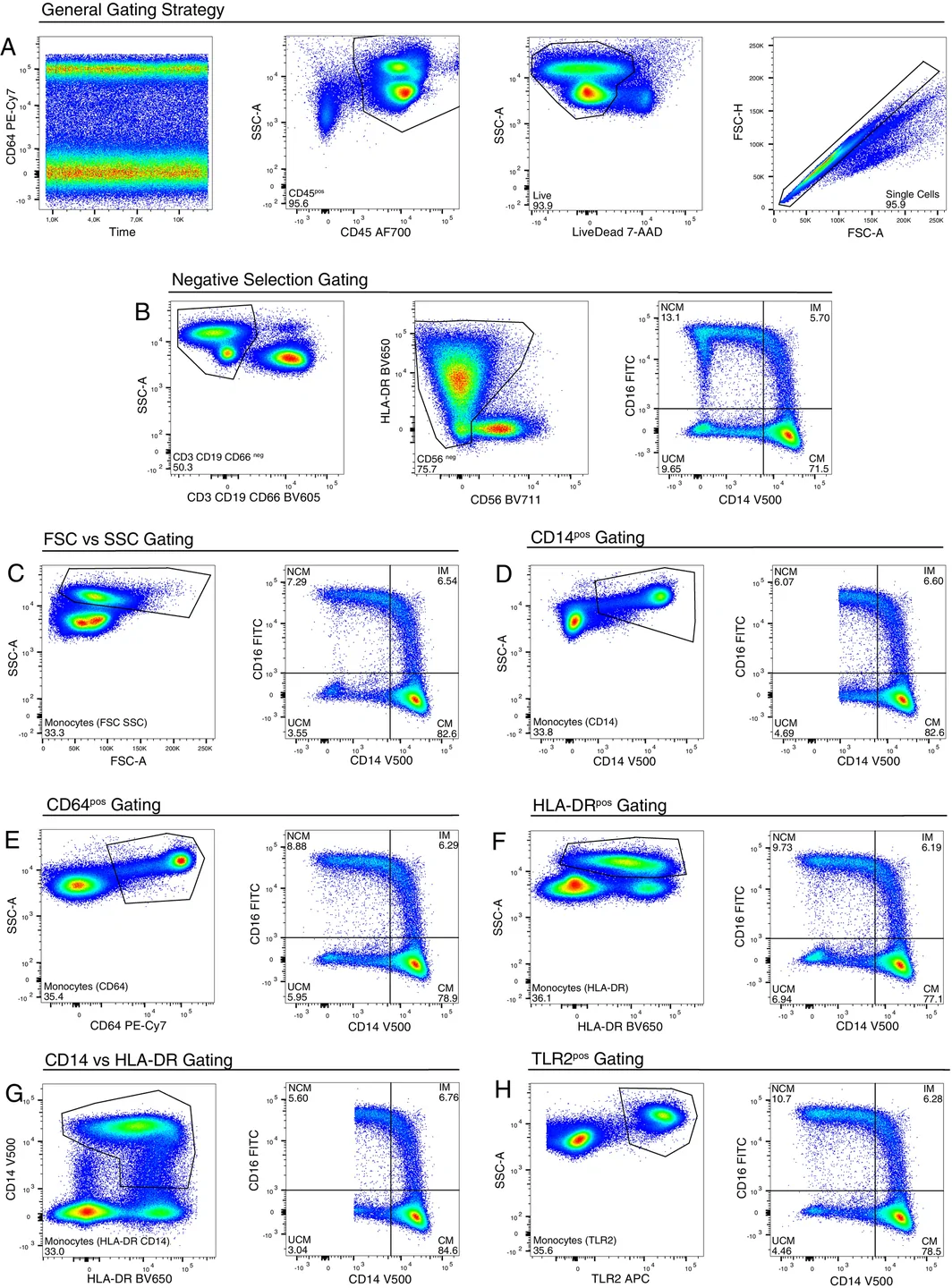
Gating strategies. (A) General gating strategy applied to identify the CD45^pos^ Live Single cell population, which was subsequently used in the respective gating strategies to define ‘all monocytes’ as shown in (B–G). (B) Negative selection gating strategy defining the monocyte population based on exclusion of CD3^pos^, CD19^pos^, CD66^pos^ and CD56^pos^ cells. (C) Forward scatter (FSC) versus side scatter (SSC) gating strategy defining the monocyte population based solely on light scatter signals. (D–H) Positive selection gating strategies defining the monocyte population based on expression of CD14 (D), CD64 (E), HLA‐DR (F), CD14 and HLA‐DR (G) and TLR2 (H). For gating strategies in (B–H), the same gates were used to identify Classical (CD14^pos^ CD16^neg^), Intermediate (CD14^pos^ CD16^pos^), Non‐classical (CD14^dim/neg^ CD16^pos^) and Unclassified (CD14^dim/neg^ CD16^neg^) monocyte subsets.

### 2.5 Statistics

All statistical analyses were conducted using Prism 8 for Mac (GraphPad Software, San Diego, CA). Normal distribution of data was evaluated using QQ plots and the Anderson‐Darling test, and data were log‐transformed if appropriate. For comparison of results across the different gating strategies, repeated measurement one‐way ANOVA with Geisser–Greenhouse correction and Tukey's multiple comparison posttest was used in Figures 3, 4, 5. Repeated measurements were used due to paired data from the same donor. All *p* values shown are corrected *p* values, and only statistically significant *p* values (< 0.05) are shown.

**FIGURE 3:**
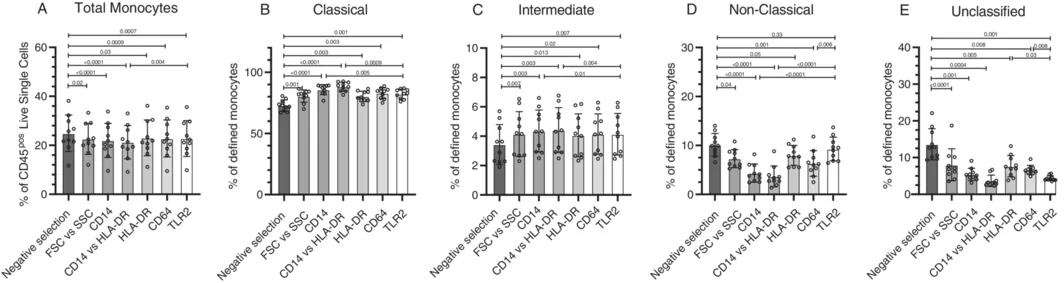
Monocyte quantification results for evaluated gating strategies. Comparison of the monocyte populations defined by the seven gating strategies: Negative selection, FSC versus SSC, CD14^pos^, CD64^pos^, HLA‐DR^pos^, HLA‐DR versus CD14 and TLR2^pos^, in healthy donor PBMCs (*N* = 10). (A) Total monocyte population as percent of CD45^pos^ Live Single cells for the seven gating strategies. (B–E) The four monocyte subsets as percent of the total monocyte populations defined by the seven gating strategies: (B) Classical (CD14^pos^ CD16^neg^), (C) Intermediate (CD14^pos^ CD16^pos^), (D) Non‐classical (CD14^dim/neg^ CD16^pos^) and (E) Unclassified (CD14^dim/neg^ CD16^neg^). Data is presented as mean with standard deviation. *p* values are shown for comparison with Negative selection and TLR2^pos^ gating, remaining *p* values can be found in Table S2. The effect sizes between the gating strategies are shown as mean ±95% CI in Figure S3A–E.

**FIGURE 4:**
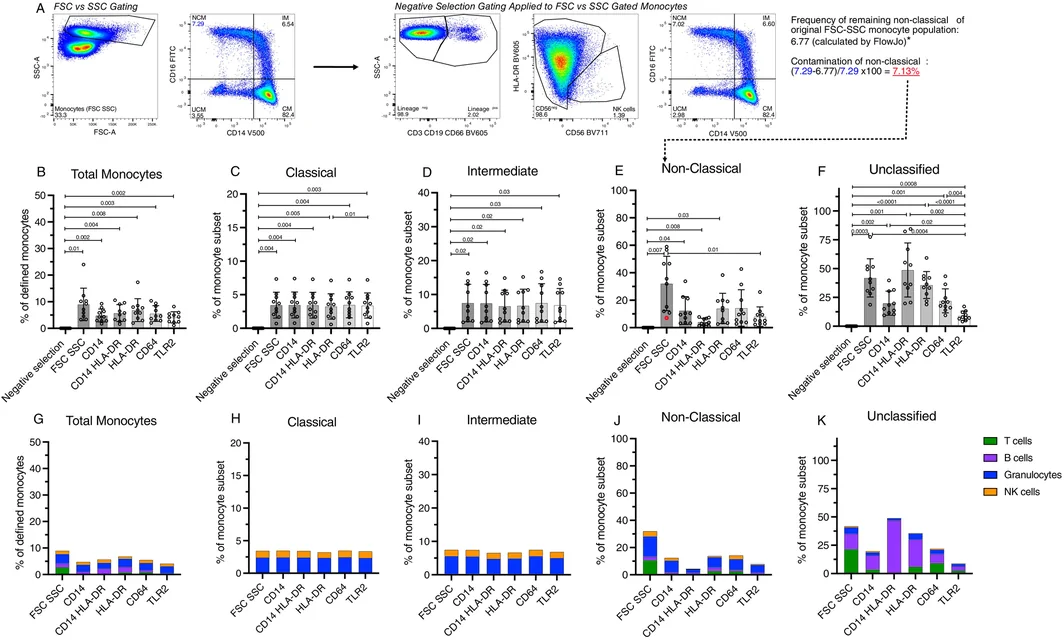
Contamination of the defined monocyte populations by non‐monocyte cells. Negative selection gating has been considered the gold standard for monocyte gating, and it was used as the reference when evaluating the other investigated monocyte identification strategies. (A) Inclusion of non‐monocytes (contamination) was investigated by applying the negative selection gating strategy on top of the investigated gating strategies, respectively. An example is shown for FSC‐SSC: Here the FSC‐SSC defined monocytes (left) were subsequently subjected to the negative selection gating strategy (right). It is seen that 7.13% of the non‐classical monocytes defined by FSC‐SSC actually expressed lineage markers, either CD3, CD19, CD66 or CD56. *Frequency of remaining (true) non‐classical monocytes from the FSC‐SSC‐defined population, after application of the negative selection gating, was calculated by FlowJo. (B–F) Data on contamination for each monocyte subset shown as the percent of the defined monocyte population, calculated as shown in (A), 0% denotes no contamination. All gating strategies showed some degree of contamination by non‐monocyte cells; FSC‐SSC the most and TLR2^pos^ the least. (G–K) Data on which non‐monocyte cells contaminated the gated subsets shown as the percent of the defined monocyte population. Non‐monocyte subsets were defined as: Granulocytes (Lin^pos^ SSC^high^), B cells (Lin^pos^ SSC^low^ HLA‐DR^pos^), T cells (Lin^pos^ SSC^low^ HLA‐DR^neg^) and NK cells (Lin^neg^ CD56^pos^). Data is shown as mean with standard deviation (SD). *p* values shown for comparison with Negative selection and TLR2^pos^ gating, remaining *p* values can be found in Table S3. The effect sizes between the gating strategies are shown as mean ±95% CI in Figure S3F–J.

**FIGURE 5:**
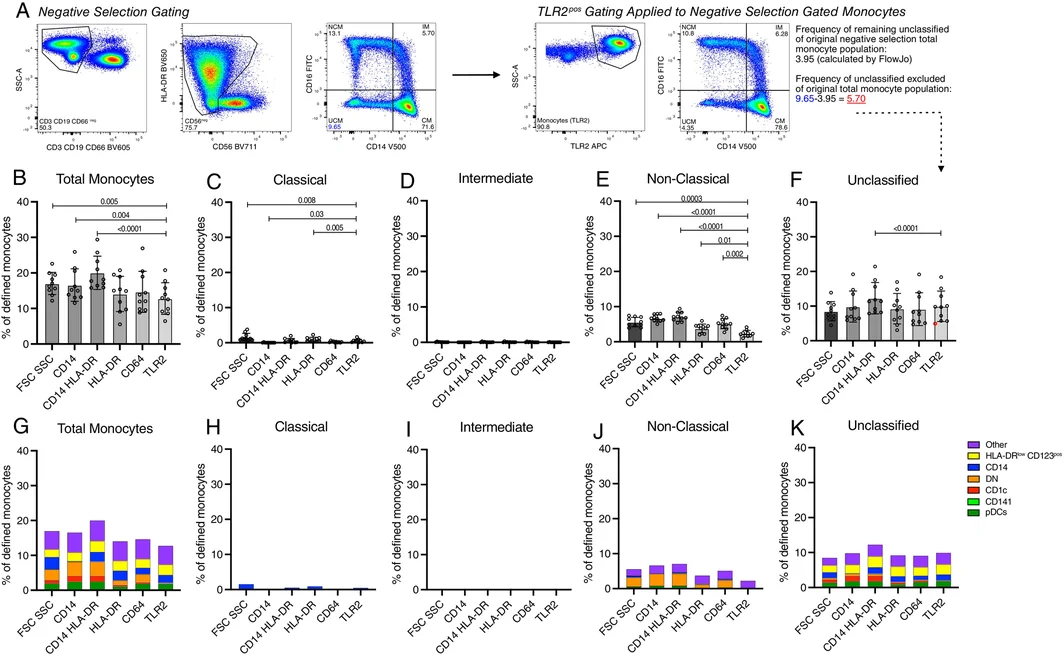
Exclusion of negative selection defined monocytes. As described, negative selection gating was used as the reference method for evaluating the other investigated monocyte identification strategies. (A) Erroneous exclusion of monocytes defined by negative selection was investigated by applying the respective gating strategies (here TLR2 as an example), to the total monocyte population defined by negative selection gating. An example is shown for TLR2^pos^ gating with a calculation for percent excluded ‘unclassified’ monocytes. It is seen that when using negative selection gating 9.65% of the total monocytes were unclassified monocytes (left). After TLR2^pos^ gating 3.95% of the original total monocytes remained in the unclassified monocyte gate (calculated by FlowJo). Thus, 5.70% of the total monocyte population was removed from the unclassified monocyte gate by TLR2^pos^ gating. (B–F) Data shown as percent exclusion of the Negative selection defined monocyte population (calculated as shown in A), 0% denotes that no monocytes were excluded. All gating strategies showed some degree of exclusion of true monocytes; CD14^pos^ HLA‐DR^pos^ the most and TLR2^pos^ the least. (G–K) Data on which cells were excluded shown as percent of the negative selection defined monocyte population. The excluded populations were defined as: pDCs (Lin^neg^ HLA‐DR^pos^ CD123^pos^), CD1c^pos^ + DCs (Lin^neg^ HLA‐DR^pos^ CD11c^pos^ CD1c^pos^), CD141^pos^+ DCs (Lin^neg^ HLA‐DR^pos^ CD11c^pos^ CD141^pos^), double negative DCs (DN, Lin^neg^ HLA‐DR^pos^ CD11c^pos^ CD1c^neg^ CD141^neg^), CD123^pos^ HLA‐DR^neg^ (Lin^neg^ CD123^pos^ HLA‐DR^neg^), CD14^pos^ cells (Lin^neg^ CD14^pos^) and other cells (Lin^neg^ CD14^neg^ CD123^neg^ CD11c^neg^ HLA‐DR^neg^). Lin^neg^ cells are negative for CD3, CD19, CD66 and CD56. Data is shown as mean with standard deviation (SD). *p* values shown for comparison with Negative selection and TLR2^pos^ gating, remaining *p* values can be found in Table S4. The effect sizes between the gating strategies are shown as mean ±95% CI in Figure S3K–O.

## 3 Results

### 3.1 Literature Overview

As described above, 542 papers were included, revealing three main categories of gating strategies (Figure 1): (1) FSC‐SSC (33.7%), (2) negative selection (23.6%) and (3) positive selection (42.6%). For the positive selection strategies, the most frequently used strategy was CD14^pos^ gating (24.5% of all papers), while others were less frequent: HLA‐DR^pos^ CD14^pos^ (5.2%), HLA‐DR^pos^ (3.9%), CD45^pos^ (1.9%), CD64^pos^ (1.5%), CD86^pos^ (1.1%), CD33^pos^ (1.1%), CD11b^pos^ (1.1%), CD4^pos^ (1.1%) and TLR2^pos^ (0.92%). During the 9.5‐year period examined, the number of papers focused on monocytes and flow cytometry increased over time, but no clear changes in the gating strategies used were observed, except that the use of FSC‐SSC‐based gating seemed to decrease in more recent years (Figure 1B,C). Thus, this overview highlights substantial methodological variation in the literature, and despite negative selection gating being considered the gold standard by several researchers [2, 23, 24], fewer than 25% of papers used this strategy.

Importantly, to our knowledge, no previous study has performed a meticulous comparison of negative selection, light‐scatter‐based and various positive selection strategies for the identification of human monocytes by flow cytometry. Therefore, we decided to investigate seven different commonly used gating strategies and their ability to identify the entire monocyte population within healthy human PBMCs: (1) FSC‐SSC, (2) negative selection, (3) CD14^pos^, (4) CD64^pos^, (5) HLA‐DR^pos^, (6) HLA‐DR^pos^ CD14^pos^ and (7) TLR2^pos^.

In designing the negative selection gating strategy, the most commonly used markers for PBMC samples were included in the panel: CD3, CD19, CD56 and CD66. This was based on our literature review, where the most commonly excluded subsets were T cells (96%), B cells (91%), NK cells (64%) and Granulocytes (64%), while any subset of DCs was excluded in only 0.3% of papers.

### 3.2 Comparison of Seven Strategies for Human Monocyte Identification

#### 3.2.1 Gating Strategies

Representative examples of the used gating strategies are shown in Figure 2. The general gating strategy (Figure 2A) was used to identify the CD45^pos^ Live Single cell population, which was then used in the subsequent gating strategies shown in Figure 2B–H. Rectangular monocyte subset gates were used, in accordance with Zawada et al., showing no significant differences between rectangular and trapezoid monocyte gates [25].

#### 3.2.2 Total Monocyte Count

When comparing total monocyte counts, as a fraction of CD45^pos^ Live Single cells, between the seven gating strategies (Figures 3A, S3, Table S2), negative selection showed a significantly higher fraction of total monocytes than the other gating strategies (e.g., negative selection (24.9% [19.6; 30.2] vs. TLR2^pos^ 22.8% [28.0; 17.5] [*p* < 0.05])).

#### 3.2.3 Monocyte Subsets: Classical, Intermediate, Non‐Classical and ‘Unclassified’

The fraction of classical monocytes (CD14^pos^ CD16^neg^) was significantly higher for all gating strategies compared to negative selection (e.g., negative selection 72.8% [69.5; 76.1], vs. TLR2 82.3% [79.5; 85.0], all *p* < 0.01, Figure 3B). The same was observed for the fraction of intermediate monocytes (CD14^pos^ CD16^pos^, all *p* < 0.05, Figure 3C), albeit with lower statistical significance. The non‐classical monocytes (CD14^dim/neg^ CD16^pos^) displayed the largest variation between gating strategies with the lowest fractions for CD14^pos^ HLA‐DR^pos^ gating (3.68% [2.14; 5.21]), which was significantly lower than negative selection (10.1% [8.47; 11.8] *p* < 0.0001). Here, only the TLR2^pos^ based results were comparable to negative selection results (9.27% [7.54; 11.0], Figure 3D).

The fraction of ‘unclassified’ monocytes (CD14^dim/neg^ CD16^neg^) defined by negative selection gating (13.6% [10.6; 16.7]) was significantly higher compared to the other gating strategies (e.g., TLR2^pos^ 4.34% [3.86; 4.83], Figure 3E).

#### 3.2.4 Erroneous Inclusion of ‘Non‐Monocytes’ and Exclusion of ‘True Monocytes’ as Defined by Negative Selection

As mentioned above, negative selection gating was used as the ‘reference standard’ for monocyte gating in the present work. Consequently, negative selection was used as the comparator to evaluate the degree of contamination by non‐monocytes and the erroneous exclusion of ‘true monocytes’ across the investigated gating strategies. The gating strategy used to define non‐monocytes (T cells, B cells, NK cells and granulocytes) is shown in Figure S2.

*Contamination by non‐monocytes* was evaluated by applying the negative selection gating strategy on top of the FSC‐SSC, CD14^pos^, CD64^pos^, HLA‐DR^pos^, CD14^pos^‐HLA‐DR^pos^ and TLR2^pos^ defined monocyte populations, respectively (Figure 4A–F). In this context, the degree of contamination was defined as the percentage of excluded cells after applying negative selection gating, for example, for the FSC‐SSC total monocyte population, ~7% of the cells were excluded by the negative selection gating (Figure 4B). No contamination would be shown as 0%.

The contamination of the total monocyte population, classical and intermediate monocytes was significant for all gating strategies (*p* < 0.05, Figures 4B–D, S3, Table S3). Non‐classical monocytes were significantly contaminated in FSC‐SSC, CD14^pos^, HLA‐DR^pos^ and HLA‐DR^pos^CD14^pos^ gating, where FSC‐SSC had the highest contamination, but for TLR2^pos^ and CD64^pos^ the level of contamination was not statistically significant (Figure 4E). For the ‘unclassified’ monocyte population, contamination was especially prevalent in HLA‐DR^pos^CD14^pos^, HLA‐DR^pos^ and FSC‐SSC gating (*p* < 0.001, Figure 4F). The distribution of contaminating cells (T cells, B cells, granulocytes and NK cells) for the different monocyte populations is shown in Figure 4G–K.

*Exclusion of true monocytes (as defined by negative‐selection)* was evaluated by applying CD14^pos^, CD64^pos^, FSC‐SSC, HLA‐DR^pos^, HLA‐DR^pos^CD14^pos^ or TLR2^pos^ gating on top of the monocyte population defined by negative selection gating (see Figures 5A, S3, Table S4). The degree of erroneous exclusion is shown as percent excluded cells, meaning no exclusion is shown as 0%.

When comparing the total monocyte populations, the gating strategies excluded between 12.8% and 20.0% of the total monocyte population compared to negative selection (Figure 5B). Between 0.02% and 1.6% of the monocytes excluded fell into the classical and intermediate monocyte gates (Figure 5C,D), while between 2.3% and 7.1% of these excluded monocytes belonged to the non‐classical subset, where TLR2^pos^ had the lowest, and CD14^pos^ and HLA‐DR^pos^CD14^pos^ gating had the highest degree of exclusion (Figure 5E). All six gating strategies showed a high degree of exclusion of ‘unclassified’ monocytes compared with negative selection, which constituted between 8.5% and 12.3% of the total negative‐selection‐defined monocytes (*p* < 0.0001, Figure 5F).

When examining these excluded true monocytes (as defined by negative selection gating), many cells were actually DC subsets (pDCs or CD1c^pos^ mDCs or CD141^pos^ mDCs), HLA‐DR^low^ CD123^pos^ (most likely basophils) or an all‐marker negative population (CD3, CD19, CD66, CD56, TLR2, CD14, CD16). Thus, only 2.2% (0.95; 3.4) of true monocytes were excluded on average, mostly non‐classical monocytes. The cells excluded by TLR2^pos^ gating appeared to be non‐monocyte subsets that were falsely included in negative‐selection‐gated ‘reference standard’ monocytes (Figure 5G). Thus, these data may call into question the view that negative selection gating strategy is the best‐practice standard for identification of human monocytes by flow cytometry and highlight that it is highly dependent on the markers included in the analysis, which is discussed further below.

### 3.3 Comparison of Gating Strategies by t‐SNE Analysis

Here, *t*‐distributed stochastic neighbour embedding (t‐SNE) plots were made (using FlowJo) for data from each separate donor (*n* = 10). Monocyte subsets defined by the different gating strategies were backgated onto the t‐SNE plots (Figure 6A). The classical, intermediate and non‐classical monocytes identified by different gating strategies showed the same localisation, whereas the ‘unclassified’ monocyte population displayed larger variation in the localisation on the t‐SNE plot.

**FIGURE 6:**
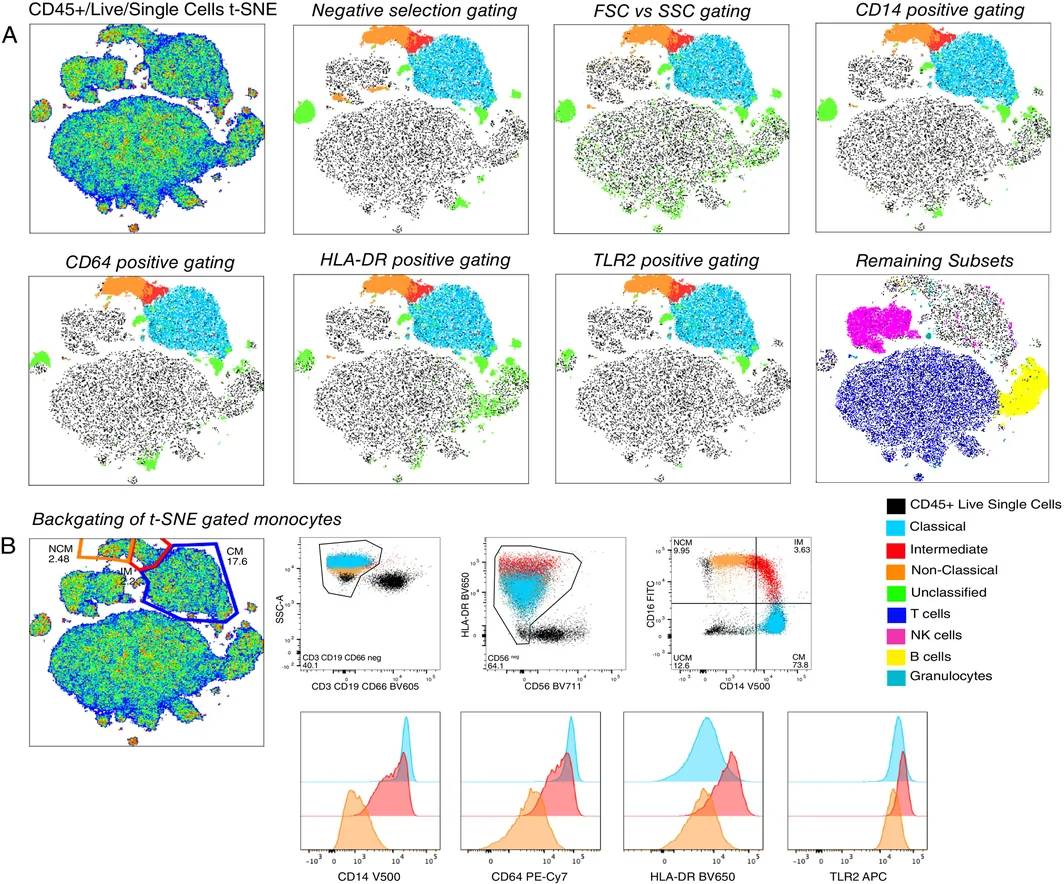
Comparison of gating strategies by t‐SNE analysis. (A) t‐SNE analysis made from the CD45^pos^ live single cells. The monocyte subsets defined by Negative selection, CD14^pos^, FSC‐SSC, CD64^pos^, HLA‐DR^pos^ and TLR2^pos^ gating strategies, respectively, were backgated onto the t‐SNE plot. (B) Classical, Intermediate and Non‐classical monocytes were gated directly on the t‐SNE plot and then backgated to the negative selection gating strategy. Histograms for Classical, Intermediate and Non‐classical monocyte expression of CD14, CD64, HLA‐DR and TLR2. Overall, it is seen that TLR2^pos^ gating included the least contaminating non‐monocytes and all backgated monocyte subsets expressed high levels of TLR2. Non‐classical monocytes expressed less CD14, CD64 and HLA‐DR compared to Intermediate and Classical, thus demonstrating that these markers are not optimal for identification of all monocyte subsets. Due to layout considerations, CD14‐HLA‐DR gating is not shown, but showed results as for HLA‐DR.

To evaluate the original gate placement (CD14 vs. CD16), classical, intermediate and non‐classical monocytes were gated directly on the t‐SNE plots, and then backgated onto the negative selection gating strategy (Figure 6B). Further, expression of the positive selection markers CD14, CD64, HLA‐DR and TLR2 is shown in histograms. These underline the disadvantage of using CD14, CD64 and HLA‐DR as positive selection markers, due to the dim expression of on non‐classical monocytes, thereby risking erroneous exclusion (as also indicated by data in Figure 4). Histograms also indicated a risk of erroneous exclusion of classical monocytes by HLA‐DR^pos^ gating. Contrarily, TLR2 showed a more uniform expression level for both classical, intermediate and non‐classical monocytes, with all subsets being clearly positive.

Heatmap t‐SNE plots showing expression levels of relevant markers supported our findings and are shown in Figure S1.

### 3.4 Comparison of Gating Strategies by Clustering Analysis

To validate our results, clustering analysis was performed using X‐shift [26] (in FlowJo) on the CD45^pos^ live single‐cell population. The quality of the clustering analysis was evaluated using both Euclid [27] and Cluster Explorer (in FlowJo). This showed adequate clustering without under‐ or over‐clustering. It also resulted in similar numbers and types of clusters for all 10 donors (a representative example is shown in Figure 7).

**FIGURE 7:**
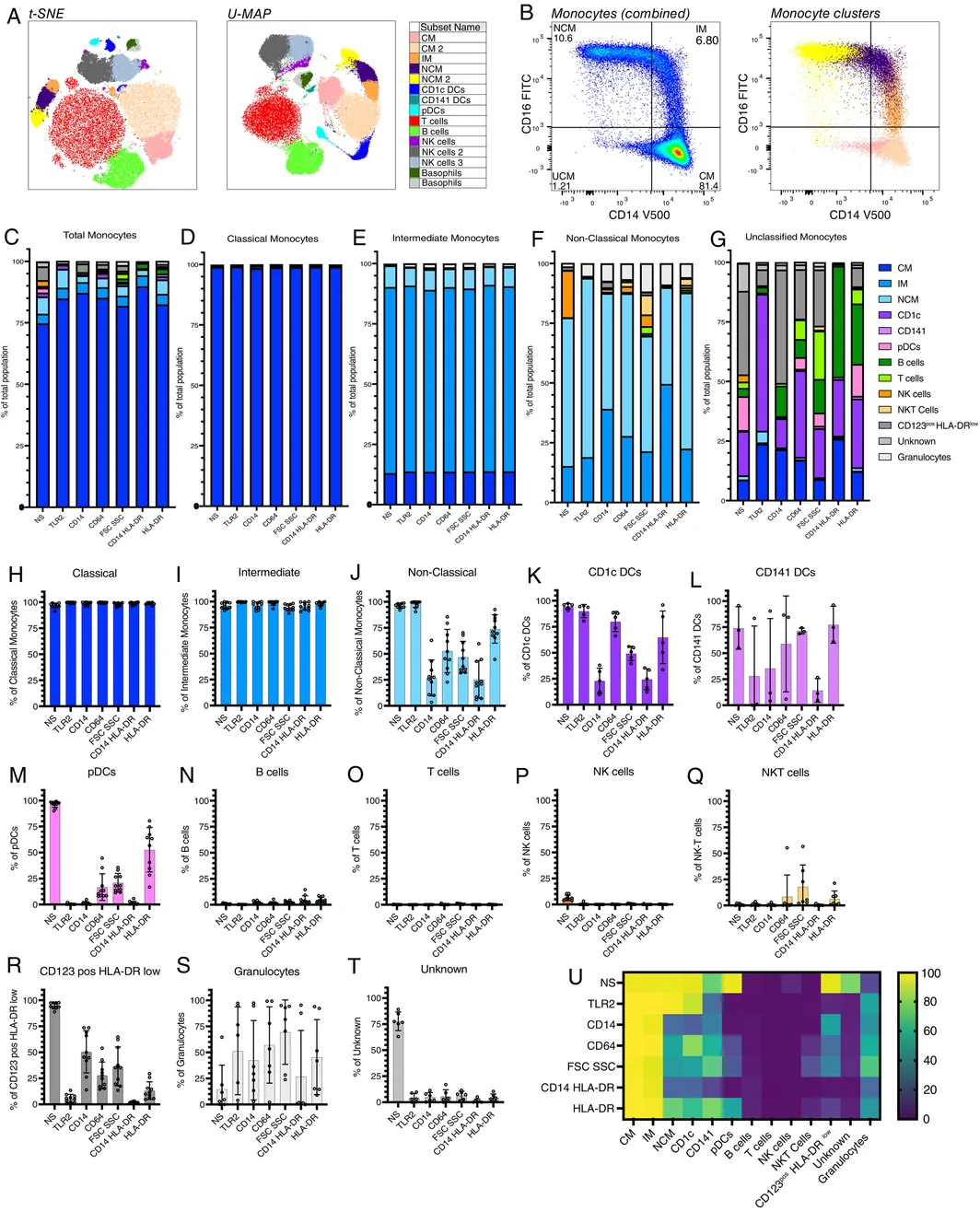
Comparison of gating strategies by clustering analysis. To more objectively examine different cell subsets, the X‐shift [26] plugin for FlowJo was used to create a clustering analysis to support the previous findings on contamination and exclusion of monocyte populations for the investigated gating strategies. Clusters were named based on expression of the markers in the panel: (1) classical monocytes (CM, CD14^pos^ CD16^neg^), (2) intermediate monocytes (IM, CD14^pos^ CD16^pos^), (3) non‐classical monocytes (NCM, CD14^dim^ CD16^pos^), (4) pDCs (CD123^pos^ HLA‐DR^pos^), (5) CD1c^pos^ DCs (CD1c^pos^ HLA‐DR^pos^ Lin^neg^), (6) CD141^pos^ DCs (CD141^pos^ HLA‐DR^pos^), (7) T cells (Lin^pos^ HLA‐DR^neg^), (8) B cells (Lin^pos^ HLA‐DR^pos^) (9) Granulocytes (Lin^pos^ CD16^pos^), (10) NK cells (Lin^neg^ CD56^pos^), (11) NK‐T cells (Lin^pos^ CD56^pos^), (12) Basophils (CD123^pos^ HLA‐DR^low^) and (13) an unknown population (negative for all included markers). (A) Representative example of the clustering analysis, with the X‐shift defined populations, shown on a t‐SNE and UMAP plot. (B) Monocyte subsets shown in a monocyte plot (CD14 vs. CD16) both combined into one population and as separate clusters. (C–G) Total monocytes (C), Classical (D), Intermediate (E), Non‐classical (F) and Unclassified (G) monocyte populations defined by the seven gating strategies shown as their cluster composition. The TLR2^pos^ gated population had the fewest non‐monocytes. (H–T) The percent of the different cell clusters included in the total monocyte population as defined by the seven different gating strategies. For example, in (J) it is seen that of all the non‐classical monocytes defined by X‐shift clustering, 50% of these cells were included in the manually gated total monocyte population defined by CD64^pos^ manual gating. Thus, 100% denotes that all cells in the cluster were included in the monocyte population of the respective manual gating method. Negative selection and TLR2^pos^ gating resulted in the fewest excluded true monocytes, especially non‐classical monocytes. (U) Heat map analysis of data from (H–T), where yellow indicate 100% of the cluster included in the gated monocytes (no exclusion), while purple indicated 0% of the cluster included in the gated monocytes. TLR2^pos^ cells also included fewest non‐monocytes, whereas negative selection showed significant contamination by non‐monocytes. Data in (H–T) is shown as mean with standard deviation.

The populations were examined using both t‐SNE and uniform manifold approximation and projection (UMAP, Figure 7A). Further, the monocyte clusters were shown both combined into one population and separately in a CD14 versus CD16 monocyte plot (Figure 7B). This highlighted significant overlap between subsets, especially intermediate and non‐classical monocytes, and that some classical monocytes had lower CD14 expression, which could lead to erroneous exclusion by traditional gating strategies that exclude CD14^neg/dim^ CD16^neg^ cells.

The generated clusters were then used to further investigate the degree of contamination (by non‐monocytic cells) of both the total monocyte population and the individual monocyte subsets, for each of the seven manual gating strategies (Figure 7C–G). This showed similar results as shown in Figure 4, with TLR2^pos^ gating yielding a low degree of contamination by non‐monocytes (< 5%), while the remaining gating strategies, including negative selection, showed significant levels of contamination, especially of the non‐classical monocytes (13.8% [5.3; 30.3]). Interestingly, the contamination seen for TLR2^pos^ gating cells was mainly by CD1c^pos^ DCs, which were located in the ‘unclassified’ monocyte population.

To further evaluate erroneous exclusion of true monocytes, we examined the fraction of generated clusters that were excluded in the manually gated monocyte populations (Figure 7H–T). Here, 100% indicates that no cells in the automated cluster were excluded from the manually gated monocyte populations. Overall, none of the classical and intermediate monocytes were excluded, while non‐classical monocytes were excluded to varying degrees by all gating strategies except for negative selection and TLR2^pos^ gating. Statistical comparisons were performed for data in Figure 7H–M (see Table S5). These findings support the results by manual gating shown in Figure 5.

Taken together, these data based on automated clustering analyses supported the results obtained by manual gating, where TLR2^pos^ gating resulted in minimal exclusion of true monocytes and limited contamination by non‐monocytes compared to the other six gating strategies.

### 3.5 Effect of TLR2 Staining on Monocyte Activation

Since it may be hypothesised that staining with TLR2 antibodies could activate monocytes and thereby affect the expression of phenotyping markers, we investigated whether TLR2 staining affected the expression of monocyte activation markers HLA‐DR, CD69, CD86 and CD163. This was investigated using two conditions: (A) a standard staining protocol with and without TLR2 staining, and (B) a protocol in which PBMCs were stained with TLR2 or stain buffer (control) for 30 min, then diluted 500× with complete medium and incubated at 37°C for 2.5 h before staining with additional antibodies. This showed no clear differences in expression levels of the investigated markers for either condition (Figure S4).

## 4 Discussion

Despite the identification of different human monocyte subsets more than 30 years ago, gating strategies used to define these cells by flow cytometry still vary considerably between studies, as highlighted here by the presented literature overview. While negative selection has been considered the ‘gold standard’ of monocyte gating by some researchers [2, 10], only 23% of the examined papers used this gating strategy. This may be due to the number of markers needed in a panel for negative selection, which may not be feasible in a setting where the panel size and costs are limiting factors.

To our knowledge, no direct comparison of several different gating strategies for flow cytometry identification of the total monocyte population within human PBMCs exists. One study has investigated the optimal monocyte gating strategies in patients with chronic myelomonocytic leukaemia (CMML); however, ‘classical monocytes’ comprised > 95% of PBMCs in these patients, and the markers differed from the commonly used monocyte markers, making direct comparison to the present results difficult [28]. Other studies used cytometry by time‐of‐flight (CyTOF) analysis to identify potential markers before applying them in traditional flow cytometry. However, the authors did not gate the total monocyte population but instead gated the three monocyte subsets separately, one by one, reducing contamination but also increasing the number of markers needed [24, 29].

Based on work by Shirk et al. [15], we identified TLR2 as a potentially useful single marker for gating of monocytes by flow cytometry. However, only limited knowledge exists regarding TLR2 as a pan‐monocytic marker (only five papers in our literature overview used TLR2).

Our investigation of seven commonly used strategies for defining the total monocyte population by flow cytometry revealed that monocyte subset fractions varied significantly between strategies, with especially CD14^pos^ and CD64^pos^ gating strategies having a significantly higher fraction of classical monocytes and a lower fraction of non‐classical monocytes, highlighting that these gating strategies *excluded* non‐classical monocytes. FSC‐SSC gating showed a lower fraction of classical monocytes and a higher fraction of non‐classical and ‘unclassified’ monocytes, indicating contamination by non‐monocytes. Importantly, TLR2^pos^ gating exhibited results that closely mirrored those of negative selection gating, characterised by minimal contamination by non‐monocytes and low exclusion of ‘true monocytes’. The results by manual gating were supported by more objective t‐SNE‐ and clustering‐based analysis. Further, clustering analysis showed that TLR2^pos^ gating had the highest percentage of monocytes included, and the least contamination, even compared to negative selection gating.

Specifically, when investigating *contamination*, we found inclusion of non‐monocytes by all gating strategies. This was particularly pronounced for FSC‐SSC gating, which included several non‐monocyte populations, especially in the non‐classical and ‘unclassified’ subsets. Backgating analysis revealed that the contaminating subsets mainly consisted of NK cells (CD56^pos^), granulocytes (SSC^high^ Lin^pos^), myeloid DCs (Lin^neg^ CD11c^pos^ HLA‐DR^pos^), plasmacytoid DCs (Lin^neg^ CD123^pos^ HLA‐DR^pos^) and basophils (Lin^neg^ CD123^pos^ HLA‐DR^neg^) [22, 30].

Overall, TLR2^pos^ gating resulted in a monocyte population with minimal contamination by non‐monocytes and the least exclusion of true monocytes, as defined by the defined ‘reference standard’ (negative selection). However, the quality of the negative selection gating strategies greatly depends on the markers included. In the present panel, based on Mair et al. 2018 [22], only CD3, CD19, CD56 and CD66 were included, while additional markers would be necessary to properly exclude, for example, DCs and basophils [22, 24].

Notably, the recent OMIP by Hally et al. [31] combined both features of negative and positive selection for identification and characterisation of monocytes and monocyte subsets. The 21‐marker 18‐colour spectral flow cytometer panel identifies monocytes based on negative selection gating (CD3^neg^, CD19^neg^, CD56^neg^, CD66b^neg^, CD123^neg^) and positive selection gating (TLR2^pos^). This paper addressed many of the pitfalls associated with negative selection gating and highlighted the potential of TLR2 as a pan‐monocytic marker. Still, few published reports have used TLR2 as a pan‐monocyte marker; thus, further research is needed.

The present study has some limitations. In our literature overview, papers were screened based on title and abstract; thus, some papers with gating of monocytes might have been missed. Further, some gating strategies are difficult to categorise as either strictly positive or negative selection, since they combine elements of both. In such cases, the papers were categorised based on the dominant gating strategy used.

Furthermore, the present study was performed on anonymised healthy blood donors from the local blood bank, meaning their characteristics were unknown. However, previous papers have described the Danish blood donor population as 54.6% female, with a median age of 36 years (IQR, 24–48), while the males were slightly older (39 years [28–50]) [32]. Thus, these donors may be relatively young compared with many diseased populations. However, a recent study on the SENEX cohort showed that TLR2 expression on monocytes was stable with age and frailty status [33]. Further, data from our previous study investigating CD163 expression on human monocytes in patients with multiple myeloma and MGUS showed similar TLR2 expression levels in both healthy controls and patients (data not shown in the paper) [34]. Still, the number of studies investigating TLR2 expression in relation to disease status remains limited, and further studies are needed in different pathological and inflammatory conditions.

In a clinical setting, many analyses would be performed on whole blood instead of PBMCs. Here, TLR2 expression in whole blood would need to be investigated further, as such data are not currently available to the best of our knowledge. However, we expect it to work in this setting as well.

Finally, when isolating monocytes for ex vivo cultures, it is well known that the isolation method strongly impacts the cells due to factors such as antibody‐induced activation during isolation [35]. Here, we showed that TLR2 staining had no significant impact on the expression of CD69, CD86, CD163 and HLA‐DR.

Based on the data presented here, TLR2 was the most promising single marker for defining the total monocyte population in healthy human PBMC samples, and it was effective in avoiding both erroneous exclusion of true monocytes and contamination by non‐monocytes. TLR2 could be used as a pan‐monocyte marker in panels where a good negative selection is not possible. In larger panels with the necessary exclusion markers, negative selection may still be feasible, but, as demonstrated here, caution should be taken to ensure that contamination by non‐monocytes is not present.

Thus, in general, we recommend that the flow cytometry community consider adapting to more uniform gating strategies for analysis of monocytes, and more specifically to consider the TLR2‐based strategy for future studies, which could increase the comparability between studies.

## Author Contributions

Conception: Morten Nørgaard Andersen. Study design: Mie Wolff Kristensen and Morten Nørgaard Andersen. Acquisition of data: Mie Wolff Kristensen, Katrine Skjøt Harbo, and Morten Nørgaard Andersen. Analysis and interpretation of data: Mie Wolff Kristensen, Thomas Vorup‐Jensen, Marianne Hokland and Morten Nørgaard Andersen. Drafting the article: Mie Wolff Kristensen and Morten Nørgaard Andersen. All authors revised the manuscript critically and approved the version to be published.

## Funding

This work was supported by the Department of Biomedicine, Aarhus University, The Danish Cancer Society (R302‐A17524), the Central Denmark Region Health Research Foundation, the Independent Research Fund Denmark (1030‐00069B), the The Novo Nordisk Foundation (NNF22OC0079892) and the Memorial Foundation of Eva and Henry Frænkel.

## Conflicts of Interest

The authors declare no conflicts of interest.

## Supporting information


**Figure S1:** tSNE plot heatmaps with expression levels of monocyte markers.
**Figure S2:** Gating strategy for identification of T cells, B cells, Granulocytes and NK cells used in the t‐SNE overlay.
**Figure S3:** Mean difference between different gating strategies.
**Figure S4:** Effect of TLR2 staining on monocyte activation markers.

**Table S1:** Overview of flow cytometry antibodies.
**Table S2:** Subset comparison of data shown in Figure 3.
**Table S3:** Comparison of ‘contamination’ between different gating strategies.
**Table S4:** Comparison of ‘erroneous exclusion’ between gating strategies.
**Table S5:** Comparison of contamination and erroneous exclusion of cell clusters between gating strategies.

## Data Availability

The data that support the findings of this study are available from the corresponding author upon reasonable request.
