# Supplementary material for: Evaluation of Methods for Identifying Human Monocytes by Flow Cytometry: TLR2 May be an Optimal Marker
Source: Immunology. 2026 Sep 7;179(4):682–94. doi: 10.1111/imm.70195 (PMC13725438; doi:10.1111/imm.70195)
Supplement: Supplementary file 1 [file IMM-179-682-s001.pdf]

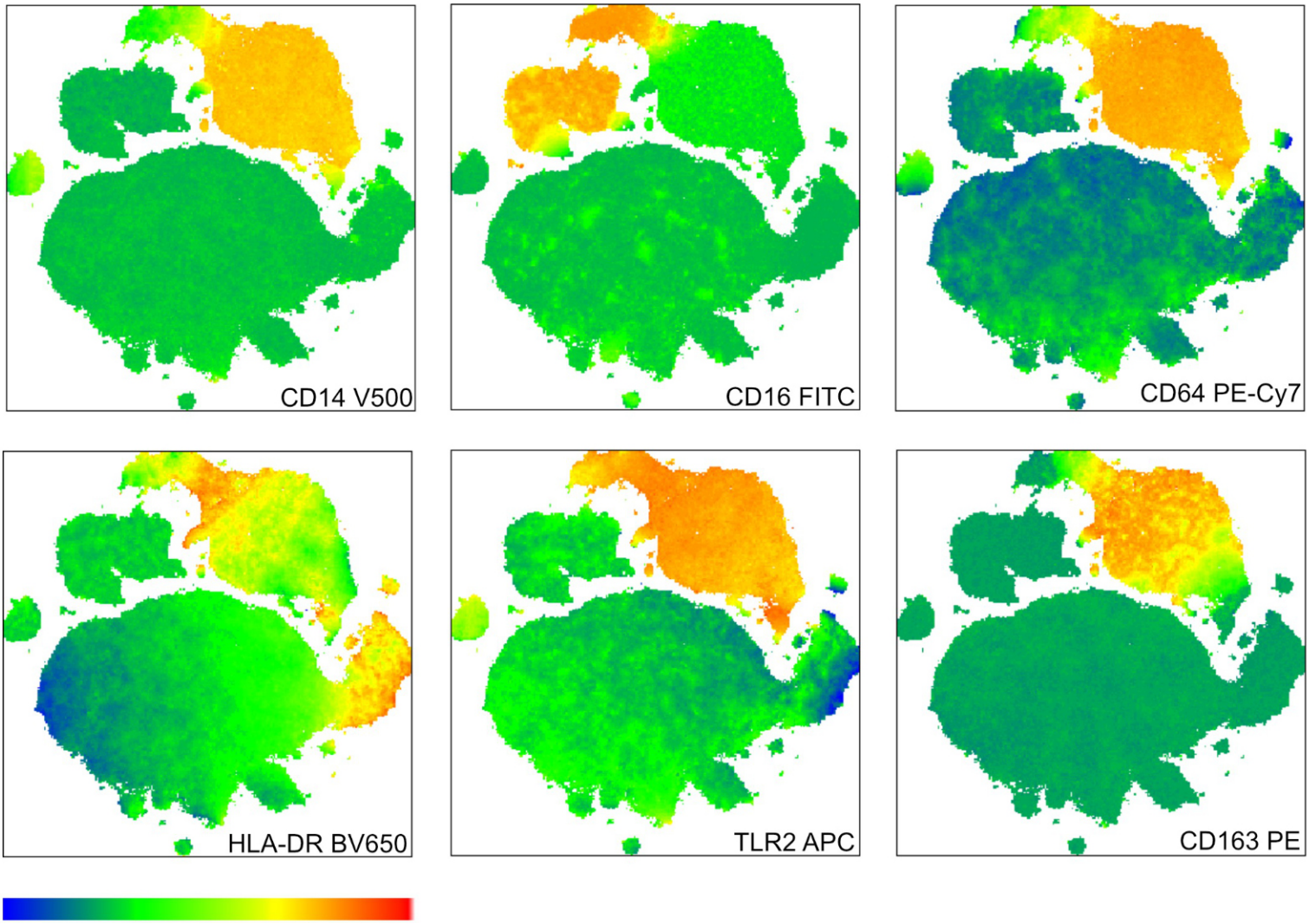

Supplementary Figure 1: The expression of the monocyte markers shown as heatmap analysis on t-SNE plot of CD45pos / Live / Single Cells (made in FlowJo). See Figure 6 for distribution of gated total monocytes and monocyte subsets by gating strategy. Blue indicate the lowest expression while red indicate the highest expression of the particular marker.

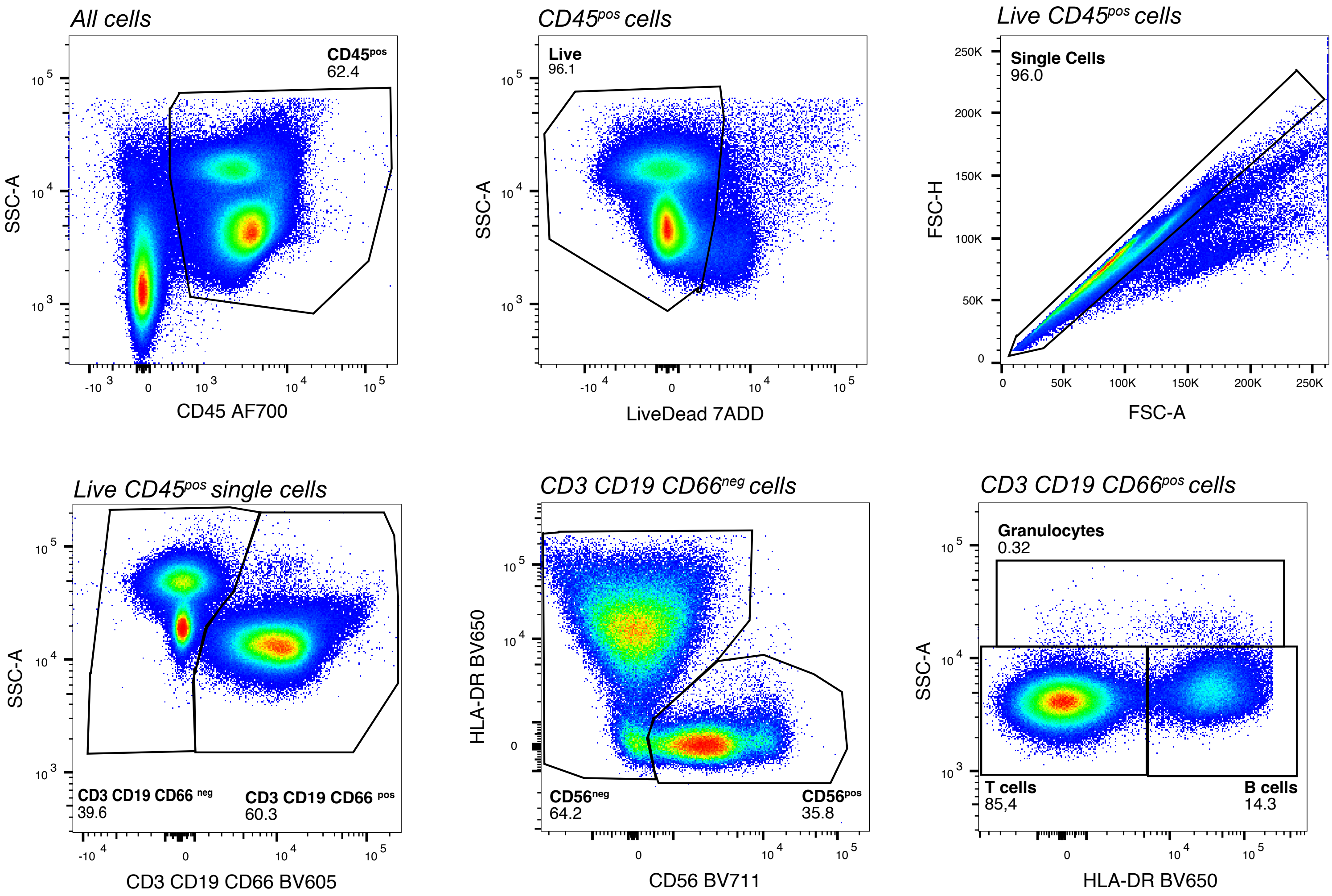

Supplementary Figure 2: Gating Strategy for Identification of T cells, B cells, Granulocytes, and NK cells used in the t-SNE overlay

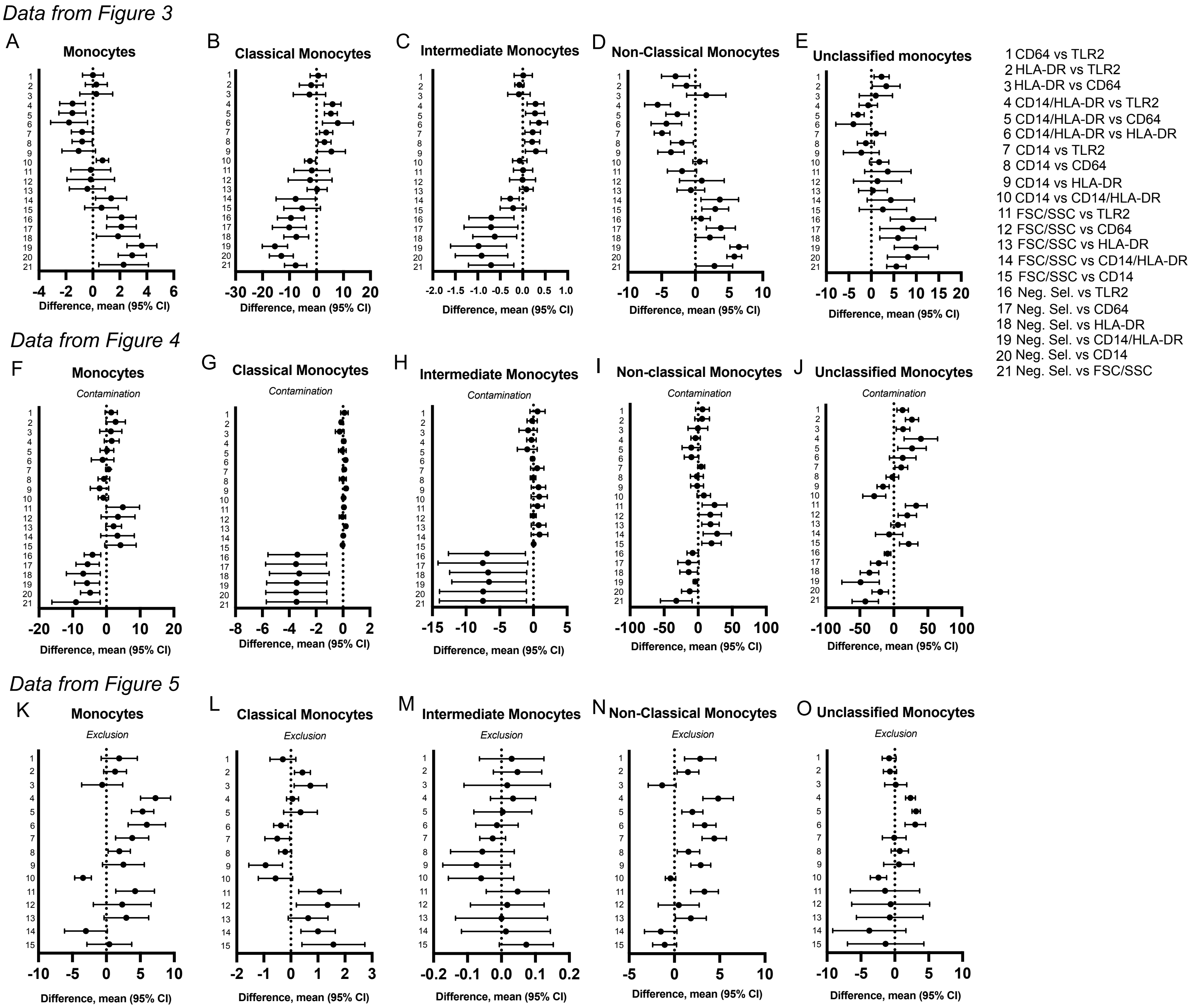

Supplementary Figure 3: Mean difference between different gating strategies.

A-E) Data from Figure 3, where differences in frequency of total monocytes (A), classical (B), Intermediate (C), non-classical (D) and unclassified (E) monocytes are shown between the different gating strategies

F-J) Data from Figure 4, where differences in contamination by non-monocytes of total monocytes (F), classical (G), Intermediate (H), non-classical (I) and unclassified (J) monocytes are shown between the different gating strategies

K-O) Data from Figure 5, where differences in exclusion of 'monocytes' from total monocytes (K), classical (L), Intermediate (M), non-classical (N) and unclassified (O) monocytes are shown between the different gating strategies

Data is shown as mean differences ± 95% confidence intervals.

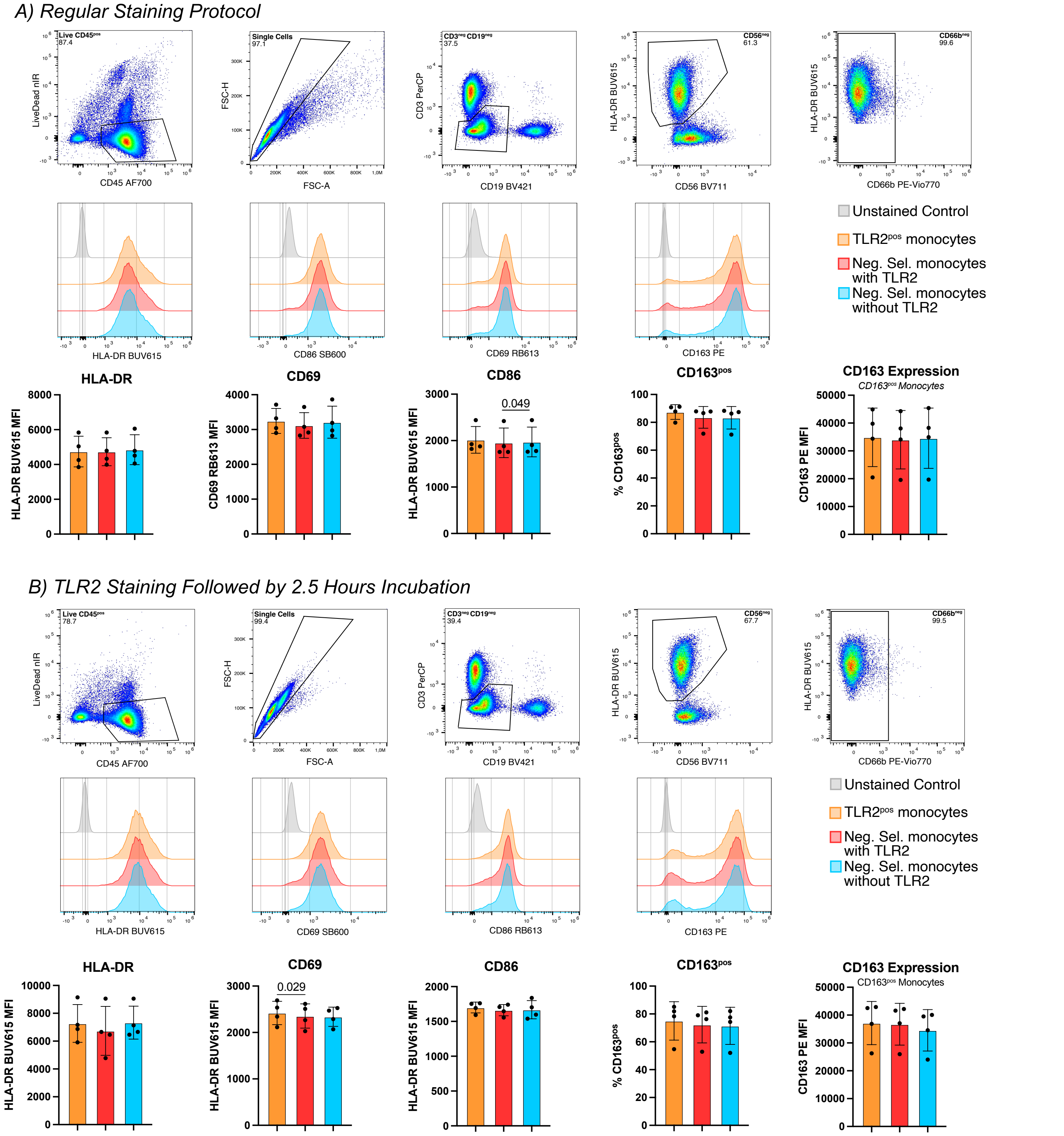

Supplementary Figure 4: Effect of TLR2 Staining on Monocyte Activation Markers

To evaluate whether TLR2 staining activates monocytes, we investigated the expression of four activation markers with/without staining with a TLR2 antibody under two conditions: A) standard staining protocol, and B) with 2.5 hours of incubation at 37°C after staining.

Here, healthy donor PBMCs (N=4) were thawed as described in the manuscript. For condition, A) the PBMCs were stained with the standard staining protocol as described. For condition B), the PBMCs were stained with TLR2 APC for 30 minutes; the antibody was diluted 500x with complete medium and was then incubated for 2.5hours at 37°C. Staining with the remaining antibodies was performed after 2.5hours for condition B using the standard procedure. For both conditions, a control without TLR2 antibody was included. The samples were run on a SONY ID7000 at the FACS Core facility, Aarhus University.

The following antibodies were used: from BD Biosciences: CD45 AF700 (Cat 560566, clone H130), CD14 V500 (Cat 562693, MP9), CD3 PerCP (Cat 345766, clone SK7), CD19 BV421 (Cat 562441, Clone HIB19), HLA-DR BUV615 (Cat 751142, Clone G46-6), CD56 BV711 (Cat 740781, Clone B159), CD16 FITC (Cat 347523, Clone NKP15), and CD69 RB613 (Cat 571125, Clone FN50). From Invitrogen, CD86 SB600 (Cat 63-0869-42, Clone IT2.2), TLR2 APC (17-9922-42, Clone TLR2.1) and LiveDead near infrared (Cat L34976). From Miltenyi, CD66b PE-Vio770 (Cat 130-119-808, Clone REA306). From IQ Products (Cat: IQP-57OR, Clone MAC2-158).

A+B) The negative selection gating used to define the total monocyte population is shown for a representative donor. Histograms showing expression of HLA-DR, CD69, CD86, and CD163 are shown for TLR2^{pos} monocytes (orange), and for negative selection-gated monocytes with (red) and without TLR2 staining (blue). Column diagrams display the median fluorescence intensity (MFI) of HLA-DR, CD69, and CD86 on the total monocyte population, as well as the frequency of CD163^{pos} cells and the CD163^{pos} MFI of CD163^{pos} monocytes. For all data, normal and lognormal distributions were evaluated, and if the data had a lognormal distribution, the data were transformed before statistical analysis was performed. One-way ANOVA for repeated measurements was performed, and a p-value of <0.05 was considered statistically significant. Tukey's post analysis was performed to account for multiple testing.

The only significant difference between staining with and without TLR2 antibody was found for the standard staining procedure (no post-staining incubation) for CD86, where the effect size was only 18.3 (SE 4.2), with mean MFIs of 1,971 vs.1,953, thus showing very small differences that we do not consider of biological relevance.

Overall, we conclude that staining with a TLR2 antibody did not lead to changes in the investigated activation markers under the investigated conditions.
