# Supplementary material for: Evaluation of Methods for Identifying Human Monocytes by Flow Cytometry: TLR2 May be an Optimal Marker
Source: Immunology. 2026 Sep 7;179(4):682–94. doi: 10.1111/imm.70195 (PMC13725438; doi:10.1111/imm.70195)
Supplement: Supplementary file 2 [file IMM-179-682-s002.docx]

| **Specificity** | **Purpose** | **Conjugate** | **Manufacturer** | **Clone** | **Cat** | **Concentration (µg/mL)** |
| --- | --- | --- | --- | --- | --- | --- |
| CD141 | mDCs | BV421 | BD Biosciences | 1A4 | 565321 | 0.125 |
| CD14 | Monocytes | V500 | BD Biosciences | MøP9 | 562693 | 3.00 |
| CD3 | T cells | BV605 | BD Biosciences | UCHT1 | 583219 | 0.50 |
| CD19 | B cells | BV605 | BD Biosciences | HIB19 | 740394 | 0.06 |
| CD66 | Granulocytes | BV605 | BD Biosciences | B1.1 | 740423 | 0.04 |
| HLA-DR | Monocytes & DCs | BV650 | BD Biosciences | G46-6 | 564231 | 0.50 |
| CD56 | NK cells | BV711 | BD Biosciences | B159 | 740781 | 0.50 |
| CD16 | Monocytes | FITC | BD Biosciences | 3G8 | 555406 | 1.50 |
| CD1c | mDCs | PerCP-eF710 | Invitrogen | L161 | 46-0015-42 | 0.06 |
| CD163 | Marker of Interest | PE | IQ Products | MAC2-158 | IQP-570R | 0.25 |
| CD11c | mDCs | PE-CF594 | BD Biosciences | B-Ly6 | 564920 | 0.30 |
| CD64 | Monocytes | PE-Cy7 | Biolegend | 22 | B06025 | 0.375 |
| TLR2 | Monocytes | APC | Miltenyi Biotec | REA109 | 130-120-052 | 1.25 |
| CD45 | Leukocytes | AF700 | BD Biosciences | HI30 | 560566 | 0.10 |
| CD123 | pDCs | APC-eF780 | Invtrogen | 6G6 | 47-1239-42 | 0.150 |
| LiveDead | Live cells | 7-AAD | BD Biosciences |  |  |  |

**Supplementary Table 1**: Overview of Flow Cytometry Antibodies

| **A Monocytes** | **Neg. Sel.** | **FSC SSC** | **CD14** | **CD14 HLA-DR** | **HLA-DR** | **CD64** | **TLR2** |
| --- | --- | --- | --- | --- | --- | --- | --- |
| **Neg. Sel.** |  | 0.0146 | <0.0001 | <0.0001 | 0.0215 | 0.0007 | 0.0006 |
| **FSC SSC** |  |  | n.s. (0.52) | 0.0201 | n.s. (0.89) | n.s. (0.99) | n.s. (0.99) |
| **CD14** |  |  |  | 0.0031 | n.s. (0.11) | 0.0356 | n.s. (0.052) |
| **CD14 HLA-DR** |  |  |  |  | 0.0114 | 0.0042 | 0.0028 |
| **HLA-DR** |  |  |  |  |  | n.s. (0.98) | n.s. (0.88) |
| **CD64** |  |  |  |  |  |  | n.s. (>0.99) |
| **TLR2** |  |  |  |  |  |  |  |
|  |  |  |  |  |  |  |  |
| **B CM** | **Neg. Sel.** | **FSC SSC** | **CD14** | **CD14 HLA-DR** | **HLA-DR** | **CD64** | **TLR2** |
| **Neg. Sel.** |  | 0.0011 | <0.0001 | <0.0001 | 0.0029 | 0.0030 | 0.0011 |
| **FSC SSC** |  |  | n.s. (0.11) | 0.0291 | n.s. (0.82) | n.s. (0.77) | n.s. (0.77) |
| **CD14** |  |  |  | 0.0177 | 0.0291 | 0.0172 | 0.0049 |
| **CD14 HLA-DR** |  |  |  |  | 0.0074 | 0.0003 | 0.0009 |
| **HLA-DR** |  |  |  |  |  | n.s. (0.58) | n.s. (0.58) |
| **CD64** |  |  |  |  |  |  | n.s. (0.77) |
| **TLR2** |  |  |  |  |  |  |  |
|  |  |  |  |  |  |  |  |
| **C IM** | **Neg. Sel.** | **FSC SSC** | **CD14** | **CD14 HLA-DR** | **HLA-DR** | **CD64** | **TLR2** |
| **Neg. Sel.** |  | 0.0068 | 0.0031 | 0.003 | 0.0125 | 0.02 | 0.0073 |
| **FSC SSC** |  |  | n.s. (0.19) | 0.007 | n.s. (0.49) | n.s. (>0.99) | n.s. (>0.99) |
| **CD14** |  |  |  | n.s. (0.73) | 0.014 | 0.0123 | 0.0123 |
| **CD14 HLA-DR** |  |  |  |  | 0.0009 | 0.0108 | 0.0038 |
| **HLA-DR** |  |  |  |  |  | n.s. (0.86) | n.s. (0.23) |
| **CD64** |  |  |  |  |  |  | n.s. (>0.99) |
| **TLR2** |  |  |  |  |  |  |  |
|  |  |  |  |  |  |  |  |
| **D NCM** | **Neg. Sel.** | **FSC SSC** | **CD14** | **CD14 HLA-DR** | **HLA-DR** | **CD64** | **TLR2** |
| **Neg. Sel.** |  | 0.0402 | <0.0001 | <0.0001 | 0.0498 | 0.0013 | n.s. (0.33) |
| **FSC SSC** |  |  | 0.0046 | 0.0107 | n.s. (0.88) | n.s. (0.92) | 0.0078 |
| **CD14** |  |  |  | n.s. (0.30) | 0.0009 | 0.0201 | <0.0001 |
| **CD14 HLA-DR** |  |  |  |  | 0.0008 | 0.0034 | <0.0001 |
| **HLA-DR** |  |  |  |  |  | n.s. (0.43) | n.s. (0.31) |
| **CD64** |  |  |  |  |  |  | 0.0062 |
| **TLR2** |  |  |  |  |  |  |  |
|  |  |  |  |  |  |  |  |
| **E UCM** | **Neg. Sel.** | **FSC SSC** | **CD14** | **CD14 HLA-DR** | **HLA-DR** | **CD64** | **TLR2** |
| **Neg. Sel.** |  | <0.0001 | 0.0012 | 0.0004 | 0.0047 | 0.0076 | 0.001 |
| **FSC SSC** |  |  | n.s. (0.57) | n.s. (0.12) | n.s. (0.99) | n.s. (0.95) | n.s. (0.22) |
| **CD14** |  |  |  | n.s. (0.12) | n.s. (0.44) | n.s. (0.31) | n.s. (0.50) |
| **CD14 HLA-DR** |  |  |  |  | 0.0432 | 0.0003 | n.s. (0.87) |
| **HLA-DR** |  |  |  |  |  | n.s. (0.94) | 0.0338 |
| **CD64** |  |  |  |  |  |  | 0.0075 |
| **TLR2** |  |  |  |  |  |  |  |

**Supplementary Table 2: Subset Comparison of Data Shown in Figure 3**

Repeated measurement one-way ANOVA analysis with paired data from Figure 3 with adjusted p-values.

Comparison of A) frequency of total monocytes out of CD45^pos^ Live cells (R^2^ = 0.7278), B) frequency of classical monocytes out of total monocytes(CM, R^2^ = 0.7463), C) frequency of intermediate monocytes out of total monocytes (IM, R^2^ = 0.7254), D) frequency of non-classical monocytes out of total monocytes (NCM, R^2^ = 0.8027), and E) frequency of unclassified monocytes out of total monocytes (UCM, R^2^ = 0.6932) between the seven gating strategies

n.s.: non-significant

R^2^ represent the proportion of of total variance explained by the analysis.

| **A Monocytes** | **Neg. Sel.** | **FSC SSC** | **CD14** | **CD14 HLA-DR** | **HLA-DR** | **CD64** | **TLR2** |
| --- | --- | --- | --- | --- | --- | --- | --- |
| **Neg. Sel.** |  | 0.0136 | 0.0019 | 0.0035 | 0.008 | 0.0026 | 0.0019 |
| **FSC SSC** |  |  | n.s. (0.08) | n.s. (0.28) | n.s. (0.077) | n.s. (0.25) | n.s. (0.055) |
| **CD14** |  |  |  | n.s. (0.38) | n.s. (0.18) | n.s. (0.68) | n.s. (0.17) |
| **CD14 HLA-DR** |  |  |  |  | n.s. (0.85) | n.s. (>0.99) | n.s. (0.20) |
| **HLA-DR** |  |  |  |  |  | n.s. (0.75) | n.s. (0.065) |
| **CD64** |  |  |  |  |  |  | n.s. (0.14) |
| **TLR2** |  |  |  |  |  |  |  |
|  |  |  |  |  |  |  |  |
| **B CM** | **Neg. Sel.** | **FSC SSC** | **CD14** | **CD14 HLA-DR** | **HLA-DR** | **CD64** | **TLR2** |
| **Neg. Sel.** |  | 0.0037 | 0.0036 | 0.0037 | 0.005 | 0.0035 | 0.0034 |
| **FSC SSC** |  |  | n.s. (0.43) | n.s. (0.99) | 0.002 | n.s. (0.99) | n.s. (0.35) |
| **CD14** |  |  |  | n.s. (0.069) | 0.0004 | n.s. (>0.99) | n.s. (0.15) |
| **CD14 HLA-DR** |  |  |  |  | 0.0009 | n.s. (0.99) | n.s. (0.75) |
| **HLA-DR** |  |  |  |  |  | n.s. (0.19) | 0.01 |
| **CD64** |  |  |  |  |  |  | n.s. (0.78) |
| **TLR2** |  |  |  |  |  |  |  |
|  |  |  |  |  |  |  |  |
| **C IM** | **Neg. Sel.** | **FSC SSC** | **CD14** | **CD14 HLA-DR** | **HLA-DR** | **CD64** | **TLR2** |
| **Neg. Sel.** |  | 0.0218 | 0.0225 | 0.0182 | 0.0205 | 0.0260 | 0.0174 |
| **FSC SSC** |  |  | n.s. (0.92) | n.s. (0.20) | n.s. (0.21) | n.s. (>0.99) | n.s. (0.35) |
| **CD14** |  |  |  | n.s. (0.20) | n.s. (0.22) | n.s. (0.99) | n.s. (0.40) |
| **CD14 HLA-DR** |  |  |  |  | n.s. (0.65) | n.s. (0.34) | n.s. (0.70) |
| **HLA-DR** |  |  |  |  |  | n.s. (0.39) | n.s. (0.96) |
| **CD64** |  |  |  |  |  |  | n.s. (0.46) |
| **TLR2** |  |  |  |  |  |  |  |
|  |  |  |  |  |  |  |  |
| **D NCM** | **Neg. Sel.** | **FSC SSC** | **CD14** | **CD14 HLA-DR** | **HLA-DR** | **CD64** | **TLR2** |
| **Neg. Sel.** |  | 0.0073 | 0.0382 | 0.0075 | 0.0324 | n.s. (0.076) | n.s. (0.06) |
| **FSC SSC** |  |  | 0.0078 | 0.0083 | 0.0061 | 0.0319) | 0.0095 |
| **CD14** |  |  |  | n.s. (0.12) | n.s. (0.99) | n.s. (0.99) | n.s. (0.10) |
| **CD14 HLA-DR** |  |  |  |  | n.s. (0.073) | n.s. (0.17) | n.s. (0.44) |
| **HLA-DR** |  |  |  |  |  | n.s. (>0.99) | n.s. (0.45) |
| **CD64** |  |  |  |  |  |  | n.s. (0.32 |
| **TLR2** |  |  |  |  |  |  |  |
|  |  |  |  |  |  |  |  |
| **E UCM** | **Neg. Sel.** | **FSC SSC** | **CD14** | **CD14 HLA-DR** | **HLA-DR** | **CD64** | **TLR2** |
| **Neg. Sel.** |  | 0.0003 | 0.0023 | 0.0013 | <0.0001 | 0.0011 | 0.0008 |
| **FSC SSC** |  |  | 0.0025 | n.s. (0.85) | n.s. (0.39) | 0.0046 | 0.0004 |
| **CD14** |  |  |  | 0.0017 | 0.0019 | n.s. (0.98) | 0.0217 |
| **CD14 HLA-DR** |  |  |  |  | n.s. (0.27) | 0.0122 | 0.0026 |
| **HLA-DR** |  |  |  |  |  | 0.0077 | <0.0001 |
| **CD64** |  |  |  |  |  |  | 0.0036 |
| **TLR2** |  |  |  |  |  |  |  |

**Supplementary Table 3: Comparison of "Contamination" between Different Gating Strategies**

Repeated measurement one-way ANOVA analysis with paired data from Figure 4 with adjusted p-values.

Comparison of "contamination" of A) total monocytes (R^2^ = 0.6417), B) classical monocytes (CM, R^2^ = 0.7783), C) intermediate monocytes (IM, R^2^ = 0.6659), D) non-classical monocytes (NCM, R^2^ = 0.6522), and E) unclassified monocytes (UCM, R^2^ = 0.8010) between the seven gating strategies.

n.s.: non-significant

R^2^ represent the proportion of of total variance explained by the analysis.

| **A Monocytes** | **FSC SSC** | **CD14** | **CD14 HLA-DR** | **HLA-DR** | **CD64** | **TLR2** |
| --- | --- | --- | --- | --- | --- | --- |
| **FSC SSC** |  | n.s. (0.99) | n.s. (0.063) | n.s. (0.083) | n.s. (0.43) | 0.0048 |
| **CD14** |  |  | <0.0001 | n.s. (0.13) | 0.0226) | 0.0035 |
| **CD14 HLA-DR** |  |  |  | 0.0003 | <0.0001 | <0.0001 |
| **HLA-DR** |  |  |  |  | n.s. (0.98) | n.s. (0.16) |
| **CD64** |  |  |  |  |  | n.s. (0.21) |
| **TLR2** |  |  |  |  |  |  |
|  |  |  |  |  |  |  |
|  |  |  |  |  |  |  |
| **B CM** | **FSC SSC** | **CD14** | **CD14 HLA-DR** | **HLA-DR** | **CD64** | **TLR2** |
| **FSC SSC** |  | 0.0087 | 0.003 | n.s. (0.097) | 0.0211 | 0.0081 |
| **CD14** |  |  | n.s. (0.086) | 0.0045 | n.s. (0.093) | 0.0292 |
| **CD14 HLA-DR** |  |  |  | 0.0077 | n.s. (0.39) | n.s. (0.91) |
| **HLA-DR** |  |  |  |  | 0.0185 | 0.0051 |
| **CD64** |  |  |  |  |  | n.s. (0.33) |
| **TLR2** |  |  |  |  |  |  |
|  |  |  |  |  |  |  |
|  |  |  |  |  |  |  |
| **C IM** | **FSC SSC** | **CD14** | **CD14 HLA-DR** | **HLA-DR** | **CD64** | **TLR2** |
| **FSC SSC** |  | n.s. (0.074) | n.s. (0.99) | n.s. (>0.99) | n.s. (0.99) | n.s. (0.50) |
| **CD14** |  |  | n.s. (0.32) | n.s. (0.19) | n.s. (0.36) | n.s. (0.25) |
| **CD14 HLA-DR** |  |  |  | n.s. (0.97) | n.s. (>0.99) | n.s. (0.49) |
| **HLA-DR** |  |  |  |  | n.s. (0.99) | n.s. (0.26) |
| **CD64** |  |  |  |  |  | n.s. (0.86) |
| **TLR2** |  |  |  |  |  |  |
|  |  |  |  |  |  |  |
|  |  |  |  |  |  |  |
| **D NCM** | **FSC SSC** | **CD14** | **CD14 HLA-DR** | **HLA-DR** | **CD64** | **TLR2** |
| **FSC SSC** |  | n.s. (0.12) | n.s. (0.11) | 0.0352 | n.s. (0.97) | 0.0003 |
| **CD14** |  |  | n.s. (0.18) | <0.0001 | 0.0135 | <0.0001 |
| **CD14 HLA-DR** |  |  |  | <0.0001 | 0.0017 | <0.0001 |
| **HLA-DR** |  |  |  |  | n.s. (0.094) | 0.0148 |
| **CD64** |  |  |  |  |  | 0.0023 |
| **TLR2** |  |  |  |  |  |  |
|  |  |  |  |  |  |  |
|  |  |  |  |  |  |  |
| **E UCM** | **FSC SSC** | **CD14** | **CD14 HLA-DR** | **HLA-DR** | **CD64** | **TLR2** |
| **FSC SSC** |  | n.s. (0.95) | n.s. (0.23) | n.s. (0.99) | n.s. (0.99) | n.s. (0.91) |
| **CD14** |  |  | 0.0006 | n.s. (0.91) | n.s. (0.37) | n.s. (>0.99) |
| **CD14 HLA-DR** |  |  |  | 0.0006 | <0.0001 | <0.0001 |
| **HLA-DR** |  |  |  |  | n.s. (0.99) | n.s. (0.20) |
| **CD64** |  |  |  |  |  | n.s. (0.13) |
| **TLR2** |  |  |  |  |  |  |

**Supplementary Table 4: Comparison of "Erroneous Exclusion" Between Gating Strategies**

Repeated measurement one-way ANOVA analysis with paired data from Figure 5 with adjusted p-values.

Comparison of "erroneous exclusion" of A) total monocytes (R^2^ = 0.7141), B) classical monocytes (CM, R^2^ = 0.6711), C) intermediate monocytes (IM, R^2^ = 0.2074), D) non-classical monocytes (NCM, R^2^ = 0.8096), and E) unclassified monocytes (UCM, R^2^ = 0.3061) between the seven gating strategies.

The gating strategy and example can be found in Figure 5.

n.s.: non-significant

R^2^ represent the proportion of of total variance explained by the analysis.

| **CM** | **Neg. Sel.** | **FSC SSC** | **CD14** | **CD14 HLA-DR** | **HLA-DR** | **CD64** | **TLR2** |
| --- | --- | --- | --- | --- | --- | --- | --- |
| **Neg. Sel.** |  | n.s. (0.75) | n.s. (0.22) | n.s. (0.19) | n.s. (0.11) | 0.0271 | 0.032 |
| **FSC SSC** |  |  | n.s. (0.41) | n.s. (0.40) | n.s. (0.65) | 0.0243 | n.s. (0.09) |
| **CD14** |  |  |  | n.s. (>0.99) | n.s. (>0.99) | n.s. (0.56) | n.s. (0.53) |
| **CD14 HLA-DR** |  |  |  |  | n.s. (>0.99) | n.s. (0.30) | n.s. (0.21) |
| **HLA-DR** |  |  |  |  |  | 0.0383 | 0.0221 |
| **CD64** |  |  |  |  |  |  | n.s. (>0.99) |
| **TLR2** |  |  |  |  |  |  |  |
|  |  |  |  |  |  |  |  |
| **IM** | **Neg. Sel.** | **FSC SSC** | **CD14** | **CD14 HLA-DR** | **HLA-DR** | **CD64** | **TLR2** |
| **Neg. Sel.** |  | n.s. (0.60) | n.s. (>0.99) | n.s. (0.99) | n.s. (0.49) | n.s. (0.19) | 0.0303 |
| **FSC SSC** |  |  | n.s. (0.46) | n.s. (0.78) | n.s. (0.13) | 0.0017 | 0.0055 |
| **CD14** |  |  |  | n.s. (0.80) | n.s. (0.77) | n.s. (0.49) | n.s. (0.12) |
| **CD14 HLA-DR** |  |  |  |  | n.s. (0.54) | n.s. (0.24) | n.s. (0.06) |
| **HLA-DR** |  |  |  |  |  | n.s. (0.97) | n.s. (0.15) |
| **CD64** |  |  |  |  |  |  | n.s. (0.76) |
| **TLR2** |  |  |  |  |  |  |  |
|  |  |  |  |  |  |  |  |
| **NCM** | **Neg. Sel.** | **FSC SSC** | **CD14** | **CD14 HLA-DR** | **HLA-DR** | **CD64** | **TLR2** |
| **Neg. Sel.** |  | n.s. (0.08) | 0.0001 | <0.0001 | n.s. (>0.99) | n.s. (0.15) | n.s. (>0.99) |
| **FSC SSC** |  |  | n.s. (>0.99) | n.s. (0.81) | n.s. (>0.99) | n.s. (>0.99) | 0.0091 |
| **CD14** |  |  |  | n.s. (>0.99) | 0.0091 | n.s. (>0.99) | <0.0001 |
| **CD14 HLA-DR** |  |  |  |  | 0.025 | n.s. (0.48) | <0.0001 |
| **HLA-DR** |  |  |  |  |  | n.s. (>0.99) | n.s. (0.81) |
| **CD64** |  |  |  |  |  |  | 0.0194 |
| **TLR2** |  |  |  |  |  |  |  |
|  |  |  |  |  |  |  |  |
| **CD1c DCs** | **Neg. Sel.** | **FSC SSC** | **CD14** | **CD14 HLA-DR** | **HLA-DR** | **CD64** | **TLR2** |
| **Neg. Sel.** |  | 0.0024 | 0.0011 | 0.0006 | n.s. (0.33) | n.s. (0.07) | n.s. (0.78) |
| **FSC SSC** |  |  | n.s. (0.08) | n.s. (0.07) | n.s. (0.88) | n.s. (0.08) | 0.0078 |
| **CD14** |  |  |  | n.s. (0.82) | n.s. (0.05) | 0.0037 | 0.0014 |
| **CD14 HLA-DR** |  |  |  |  | n.s. (0.07) | 0.0027 | 0.0008 |
| **HLA-DR** |  |  |  |  |  | n.s. (0.85) | n.s. (0.29) |
| **CD64** |  |  |  |  |  |  | n.s. (0.48) |
| **TLR2** |  |  |  |  |  |  |  |
|  |  |  |  |  |  |  |  |
| **CD141 DCs** | **Neg. Sel.** | **FSC SSC** | **CD14** | **CD14 HLA-DR** | **HLA-DR** | **CD64** | **TLR2** |
| **Neg. Sel.** |  | n.s. (>0.99) | n.s. (>0.99) | n.s. (>0.99) | n.s. (>0.99) | n.s. (>0.99) | n.s. (>0.99) |
| **FSC SSC** |  |  | n.s. (>0.99) | n.s. (>0.99) | n.s. (>0.99) | n.s. (>0.99) | n.s. (>0.99) |
| **CD14** |  |  |  | n.s. (>0.99) | n.s. (>0.99) | n.s. (>0.99) | n.s. (>0.99) |
| **CD14 HLA-DR** |  |  |  |  | n.s. (>0.99) | n.s. (>0.99) | n.s. (>0.99) |
| **HLA-DR** |  |  |  |  |  | n.s. (>0.99) | n.s. (>0.99) |
| **CD64** |  |  |  |  |  |  | n.s. (>0.99) |
| **TLR2** |  |  |  |  |  |  |  |
|  |  |  |  |  |  |  |  |
| **pDCs** | **Neg. Sel.** | **FSC SSC** | **CD14** | **CD14 HLA-DR** | **HLA-DR** | **CD64** | **TLR2** |
| **Neg. Sel.** |  | n.s. (0.48) | <0.0001 | <0.0001 | n.s. (>0.99) | n.s. (0.08) | <0.0001 |
| **FSC SSC** |  |  | n.s. (0.24) | n.s. (0.31) | n.s. (>0.99) | n.s. (>0.99) | 0.0041 |
| **CD14** |  |  |  | n.s. (>0.99) | 0.0033 | n.s. (>0.99) | n.s. (>0.99) |
| **CD14 HLA-DR** |  |  |  |  | 0.005 | n.s. (>0.99) | n.s. (>0.99) |
| **HLA-DR** |  |  |  |  |  | n.s. (>0.99) | <0.0001 |
| **CD64** |  |  |  |  |  |  | 0.0399 |
| **TLR2** |  |  |  |  |  |  |  |

**Supplementary Table 5: Comparison of Contamination and Erroneous Exclusion of Cell Clusters Between Gating Strategies**

P-values from comparison analysis of Figure 7H-M. Clusters were defined using X-shift (in FlowJo) and exclusion monocyte clusters and contamination of DC clusters were evaluated by backgating analysis in FlowJo.

One-way ANOVA analysis of erroneous exclusion of A) classical monocyte cluster (CM, R^2^ = 0.4744), B) intermediate monocyte cluster (IM, R^2^ = 0.4477), or C) non-classical monocyte cluster (NCM, R^2^ = 0.8707). One-way ANOVA analysis of contamination of 'total monocytes' by D) CD1c dendritic cell cluster (DCs, R^2^ = 0.9014), E) CD141 DC cluster (R^2^ = 0.5500), or F) pDC cluster (R^2^ = 0.9401).

n.s. = non-significant

R^2^ represent the proportion of of total variance explained by the analysis.
